# How the “Understanding Research Evidence” Web-Based Video Series From the National Collaborating Centre for Methods and Tools Contributes to Public Health Capacity to Practice Evidence-Informed Decision Making: Mixed-Methods Evaluation

**DOI:** 10.2196/jmir.6958

**Published:** 2017-09-28

**Authors:** Linda Chan, Jeannie Mackintosh, Maureen Dobbins

**Affiliations:** ^1^ National Collaborating Centre for Methods and Tools School of Nursing McMaster University Hamilton, ON Canada

**Keywords:** public health, public health practice, evidence-based practice, capacity building, continuing education, computer-assisted instruction

## Abstract

**Background:**

The National Collaborating Centre for Methods and Tools (NCCMT) offers workshops and webinars to build public health capacity for evidence-informed decision-making. Despite positive feedback for NCCMT workshops and resources, NCCMT users found key terms used in research papers difficult to understand. The Understanding Research Evidence (URE) videos use plain language, cartoon visuals, and public health examples to explain complex research concepts. The videos are posted on the NCCMT website and YouTube channel.

**Objective:**

The first four videos in the URE web-based video series, which explained odds ratios (ORs), confidence intervals (CIs), clinical significance, and forest plots, were evaluated. The evaluation examined how the videos affected public health professionals’ practice. A mixed-methods approach was used to examine the delivery mode and the content of the videos. Specifically, the evaluation explored (1) whether the videos were effective at increasing knowledge on the four video topics, (2) whether public health professionals were satisfied with the videos, and (3) how public health professionals applied the knowledge gained from the videos in their work.

**Methods:**

A three-part evaluation was conducted to determine the effectiveness of the first four URE videos. The evaluation included a Web-based survey, telephone interviews, and pretest and posttests, which evaluated public health professionals’ experience with the videos and how the videos affected their public health work. Participants were invited to participate in this evaluation through various open access, public health email lists, through informational flyers and posters at the Canadian Public Health Association (CPHA) conference, and through targeted recruitment to NCCMT’s network.

**Results:**

In the Web-based surveys (n=46), participants achieved higher scores on the knowledge assessment questions from watching the OR (*P*=.04), CI (*P*=.04), and clinical significance (*P*=.05) videos but not the forest plot (*P*=.12) video, as compared with participants who had not watched the videos. The pretest and posttest (n=124) demonstrated that participants had a better understanding of forest plots (*P*<.001) and CIs (*P*<.001) after watching the videos. Due to small sample size numbers, there were insufficient pretest and posttest data to conduct meaningful analyses on the clinical significance and OR videos. Telephone interview participants (n=18) thought the videos’ use of animation, narration, and plain language was appropriate for people with different levels of understanding and learning styles. Participants felt that by increasing their understanding of research evidence, they could develop better interventions and design evaluations to measure the impact of public health initiatives.

**Conclusions:**

Overall, the results of the evaluation showed that watching the videos resulted in an increase in knowledge, and participants had an overall positive experience with the URE videos. With increased competence in using the best available evidence, professionals are empowered to contribute to decisions that can improve health outcomes of communities.

## Introduction

Evidence-informed decision making in public health “integrates science-based interventions with community preferences to improve the health of populations” [1]. The use of evidence can impact intervention effectiveness in public health to minimize inequities in the community [2]. To cut through the complexity of applying evidence in practice, a clear method of how to do so is needed [3].

The National Collaborating Centre for Methods and Tools (NCCMT) has developed a seven-step process to engage with evidence-informed decision making in public health. The process outlines how to use the “best available evidence from research, context, and experience to inform and improve public health policy and practice” [4]. The seven steps of the evidence-informed public health process are as follows: defining the problem, searching for evidence, appraising the evidence, synthesizing the results of the evidence, adapting the evidence to the local context, implementing the knowledge translation strategy and the intervention, and evaluating the knowledge translation strategy and the outcomes of the intervention [5]. This process guides public health professionals through the process of finding and using the best available evidence when designing a public health intervention for their local context [4]. The NCCMT delivers training workshops on the evidence-informed public health process and critical appraisal to public health professionals across Canada. Through delivering these workshops, NCCMT staff identified understanding key concepts in research literature as a barrier to engaging with evidence.

Research literacy skills are necessary to facilitate the practice of evidence-informed decision-making [6]. Along with the lack of time to find and implement evidence, the lack of confidence in interpreting research is another barrier to using evidence in practice [7-9]. Public health professionals need to have dedicated time and access to high quality continuing professional development education on technical research concepts. This training will ensure that public health professionals understand research evidence to be able to engage in evidence-informed decision making [8,10].

NCCMT’s Understanding Research Evidence (URE) video series was created to provide training on common research concepts. The URE videos were produced to be engaging and easily accessible for public health professionals. These videos are a Web-based resource that supports the practice of evidence-informed decision making in public health by building competency in interpreting research evidence. The topics in the series were chosen, based on feedback from previous workshop participants and the experience of NCCMT staff members. Communication, video production, and animation professionals were consulted in the development of the script and the creation of the videos. The first step in developing each video was writing a script. The initial draft was edited and revised substantially by JM to translate technical explanations of the concepts into plain language, while maintaining accuracy and clarity. MD has expertise on the technical aspects of the research concepts and has extensive experience training others to understand and conduct research. MD also reviewed the initial drafts to ensure the quality of the information. Throughout the editing process, the NCCMT team met regularly with a video production team and a cartoonist to discuss how best to convey the concepts visually. Narration of the final script was recorded on camera, and the cartoons were inserted. All videos in the series were produced in both English and French. The videos are available on the NCCMT website [11] and on YouTube. The first four URE videos in the series are on odds ratio (OR), clinical significance, confidence intervals (CIs), and forest plots. The videos describe the meaning of the concept and how to interpret or calculate the results. The first four videos in the URE series ranged from 3.5 min to 5.5 min.

An evaluation was conducted to examine public health professionals’ experience with the first four videos in the URE video series to explain how the videos affected their work. In evaluations of education interventions, it is important to explore new ways to understand concepts, to justify the education intervention, and to improve the delivery of the intervention [12]. This evaluation examined both the delivery mode and the content of the videos. Specifically, this evaluation explored (1) whether the videos were effective at increasing knowledge on the four video topics, (2) whether public health professionals were satisfied with the videos, and (3) how public health professionals applied the knowledge gained from the videos in their work.

## Methods

**Figure 1:**
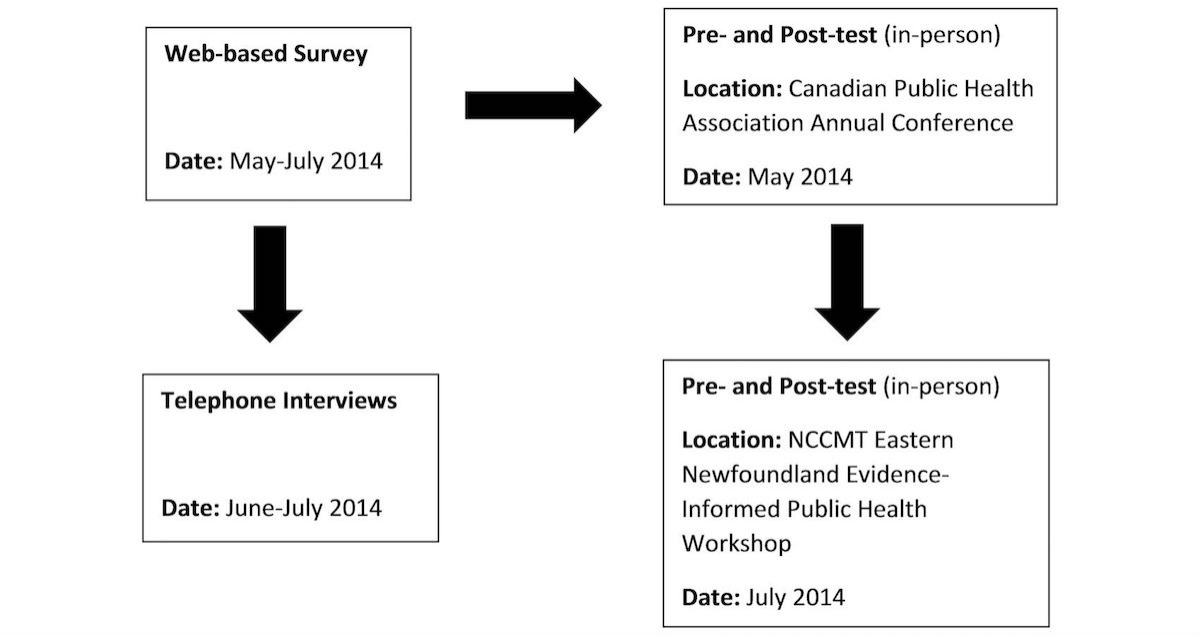
Evaluation of the Understanding Research Evidence videos: data collection.

A mixed-methods approach was used for this evaluation. A Web-based survey, pretest and posttests, and semistructured telephone interviews were conducted to capture public health professionals’ experiences with the URE videos. The data collected provided information on how to strengthen the design and delivery of the videos to maximize their utility for public health professionals. Figure 1 outlines the timeline of data collection. Both the Web-based survey and the pretest and posttests were used to collect data on the videos’ ability to increase participants’ knowledge on ORs, clinical significance, forest plots, and CIs. The Web-based survey increased the reach of this evaluation to public health professionals across Canada because of its easy accessibility. To build on the data collected from the Web-based survey, the pretest and posttests provided data using a stronger measure to assess the knowledge gained from the videos. Data from the telephone interviews complemented data from the Web-based survey and the pretest and posttests by exploring how the videos helped participants learn about research concepts and how participants used the videos in their public health work. Research ethics approval for this evaluation was obtained from the University of Toronto.

### Sample

Since the NCCMT resources are intended for public health professionals across Canada, the evaluation sample included a broad range of Canadian public health professionals. Individuals were required to have watched at least one of the videos on ORs, clinical significance, CIs, and forest plots to participate in the Web-based survey and the semistructured telephone interviews. It was assumed that individuals would have access to computers and the Internet, and have basic computer skills, because most public health professionals are required to use computers as part of their role. Participation in the Web-based survey, pretest and posttest, and telephone interviews was voluntary.

### Data Collection

#### Web-Based Survey

Invitations to participate in the Web-based survey were sent out to four different public health Web-based email lists: the NCCMT Weekly Round-Up newsletter, the Health Evidence newsletter, Community Health Networking-Works newsletter, and the Public Health Knowledge Translation Network mailing list. Medical Officers of Health across Canada were also asked to invite their staff to participate in the Web-based survey through email. Public health professionals, who self-determined that they met sample criteria, could access the open survey through a link provided in the invitation. The Web-based survey was conducted from May 28, 2014 to July 2, 2014 on Qualtrics Research Suite (Qualtrics, Salt Lake City, Utah), and it collected data on who had been using the videos, how the videos were being accessed, and assessed participants’ knowledge on ORs, CIs, forest plots, and clinical significance. A letter of information introducing the survey and the purpose of the evaluation was included in the first page of the Web-based survey. Consent to participate in the survey was provided from participants when they clicked to continue past the first page. The data collected was anonymous, and all the responses were stored on an encrypted computer. The multiple-choice knowledge assessment questions tested the participants’ ability to understand, interpret, and apply the research concepts (see Multimedia Appendix 1). The knowledge assessment questions were written by LC and vetted by MD, who has expertise on the concepts through her research and teaching commitments. The response options provided different explanations and interpretations of the research concept with only one correct answer. In order to demonstrate that they had knowledge of the concept, participants’ needed to select the correct answer. Web-based survey participants had the option to review and change their answers throughout the survey. At the end of the survey, participants had the option to enter their name for a draw to win a $50 gift card (CAD) to a Canadian bookstore.

#### Pretest and Posttest

Two sets of in-person pretest and posttests were conducted concurrent to the Web-based survey. The pretest and posttests assessed whether knowledge was gained from watching the URE videos. Four separate tests were created for each of the URE videos. The knowledge assessment questions were written in the same manner as the knowledge assessment questions in the Web-based survey (see Multimedia Appendix 1). Each test had a set of 5 to 6 multiple-choice questions that asked about key ideas necessary to understand the research concept.

The first pretest and posttest was conducted in-person at the Canadian Public Health Association (CPHA) annual conference in May 2014. Participants were recruited using informational flyers and posters at the NCCMT’s CPHA exhibition booth. A message inviting individuals to participate in a video-viewing session was also included in the NCCMT Weekly Round-Up E-newsletter. A random schedule was created for the video showings. Twelve video-viewing sessions were held for the four URE videos. Participants were blinded to this schedule, so they did not know which of the four URE videos was being shown at the start of their participation. At the video-viewing sessions, individuals were invited to complete an informed consent form, a pretest, watch one of the four URE videos, and complete a posttest.

Due to the low participation rate for the initial pretest and posttest session, another session was conducted using iClicker technology at the NCCMT’s Eastern Newfoundland evidence-informed public health and critical appraisal workshops in July 2014. Additional data was collected on the forest plot and CI videos.

#### Telephone Interview

The semistructured telephone interviews explored the following: why public health professionals watched the URE videos, whether the videos were engaging, how the videos facilitated learning, and how the knowledge gained from the videos was used in participants’ professional work. Individuals who completed the Web-based survey were invited to participate in an interview. However, this recruitment method only rendered 2 participants. To recruit additional interview participants, individuals from the NCCMT network were contacted and invited to participate in an interview. Once individuals agreed to participate in a telephone interview, any individuals who had not seen one of the URE videos were asked to watch one of the four videos prior to their scheduled interview time.

To ensure that participants provided informed consent to participate in the interview, they were emailed a letter of information about the evaluation prior to the interview. At the beginning of the interview, a brief overview of the evaluation was provided to the participants to ensure that they understood for what they were providing consent. After the overview, participants were asked to provide verbal consent to participate in the interview and to have the interview recorded.

### Data Analysis

#### Web-Based Survey

Descriptive frequencies were used to analyze demographic data. Participants were given a point for the answers they got correct in the knowledge assessment questions in the survey. A three by two chi-square (χ^2^) test was used to analyze the relationship between the videos watched and the total knowledge assessment score. All responses were reviewed to determine the completeness of each survey. Internet Protocol addresses were also reviewed to identify any potential duplicate entries from the same user. Incomplete questionnaires were included in analysis. Missing data were reported in the results section.

#### Pretest and Posttest

A paired *t*-test was used to analyze the amount of knowledge gained on each research concept before and after watching the video shown for the pretest and posttest. The Web-based survey data and pretest and posttest data were analyzed using SPSS Statistics (IBM, Chicago, Illinois).

#### Telephone Interviews

A framework analysis approach was used to analyze the telephone interview data. Interviews were transcribed and coded by LC to gain familiarity with the data. The interview transcripts were reviewed initially to identify themes about participants’ thoughts and experiences that emerged with the URE videos. The interviews were indexed iteratively by systematically reapplying codes that emerged from one interview to the entire data set. Finally, relationships between themes were explored to make connections and associations between concepts [13]. Dedoose software (SocioCultural Research Consultants, Los Angeles, California) was used for analysis of the interviews.

## Results

### Web-Based Survey: Demographic Information

Results from the Web-based survey (n=46) described the type of public health professionals who had watched the URE videos. The survey respondents spanned across Canada from British Columbia, Alberta, Saskatchewan, Manitoba, Ontario, Newfoundland, Nova Scotia, Yukon, and the Northwest Territories. Table 1 summarizes the survey respondents’ education level, amount of public health work experience, and occupation roles. Table 2 describes how respondents found out about the URE videos and where they watched the videos.

### Web-Based Survey: Comprehension of the Research Concepts

In the Web-based survey, 33% (15/46) of participants stated they had a good understanding of research concepts, and 96% (44/46) of survey participants felt that they gained knowledge from watching the videos. Despite many participants having a baseline understanding of the research concepts, results from the chi-square test showed there was an increase in the number of knowledge assessment questions that participants got right from watching the URE videos.

Table 3 summarizes the results of the Web-based survey knowledge assessment questions. For the OR, CI, and clinical significance videos, a relationship was found between having watched the URE video and a higher score on the knowledge assessment questions (OR χ^2^_2_ [N=46]=6.7, *P*=.04; CI χ^2^_2_ [N=46]=6.7, *P*=.04; clinical significance χ^2^_2_ [N=46]=6.0, *P*=.05). The results for the forest plot video were not statistically significant (forest plots χ^2^_2_ [N=46]=4.3, *P*=.12; *P*<.05). There was insufficient data to determine whether or not watching the forest plot video resulted in higher scores on the knowledge assessment questions. For the other three videos, the Pearson correlation ([OR: *r*=.348], [Clinical Significance: *r*=.286], [95% CI: *r*=.348]) indicates that there was a medium correlation between having watched the URE video and participants’ score on the knowledge assessment questions.

**Table 1:** Location, education, public health work experience, and occupation of Web-based survey participants.

Demographic		Number of participants (%)
**Province^a^**		
	British Columbia	2 (4)
	Alberta	4 (8)
	Saskatchewan	2 (4)
	Manitoba	1 (2)
	Ontario	23 (51)
	Newfoundland and Labrador	10 (22)
	Nova Scotia	1 (2)
	Yukon	1 (2)
	Northwest Territories	1 (2)
	Total	45
**Education**		
	Bachelor’s degree	23 (50)
	Master’s degree	20 (43)
	MD (Medical Doctor)	1 (2)
	Doctorate (PhD, EdD)	2 (4)
	Total	46
**Public health work experience^a^**		
	Less than 1 year	7 (15)
	1-5 years	8 (17)
	6-10 years	9 (20)
	11-15 years	11 (24)
	16-20 years	2 (4)
	21-25 years	3 (6)
	25+ years	5 (11)
	Total	45
**Occupation^a^**		
	Health promotion/Educator	8 (17)
	Dietitian/Public health nutritionist	4 (8)
	Public health nurse/Registered nurse	10 (22)
	Academic/Professor	2 (4)
	Program Evaluator	3 (6)
	Consultant/Specialist	2 (4)
	Researcher/Research analyst	6 (13)
	Other	10 (22)
	Total	45

^a^Number of participants does not equal 46 because of missing data.

**Table 2:** Accessing the URE videos.

Access to the videos		Number of participants (%)
**Learned about videos^a^**		
	NCCMT weekly round-up newsletter	2 (4)
	NCCMT website	4 (8)
	NCCMT workshops	2 (4)
	Twitter	1 (2)
	Canadian Public Health Association Conference 2014	23 (51)
	Word of mouth	10 (22)
	Total	1 (2)
**Location where videos were watched**		
	NCCMT website	35 (76)
	YouTube	3 (6)
	Training session	6 (13)
	Not sure	2 (4)
	Total	46

^a^Number of participants does not equal 46 because of missing data.

**Table 3:** Frequency tables of Web-based survey knowledge assessment scores.

Video	Knowledge assessment score	Did not watch^a^	Watched^a^	Total	*r* (*P* value)	
**Odds ratio video**						.348 (.04)
	0	4	2	6		
	1	6	18	24		
	2	2	14	16		
	Total	12	34	46		
		
**Forest plot video**						.296 (.12)
	0	4	4	8		
	1	4	12	16		
	2	3	19	22		
	Total	11	35	46		
		
**Clinical significance video**						.286 (.05)
	0	6	3	9		
	1	8	26	34		
	2	1	2	3		
	Total	15	31	46		
		
**Confidence interval video**						.348 (.04)
	0	4	2	6		
	1	6	18	24		
	2	2	14	16		
	Total	12	34	46		

^a^A correction factor was included in the χ^2^ analysis on SPSS to account for the small cell sizes in the frequency table.

**Table 4:** Pre- and posttest knowledge assessment scores.

Video	Mean difference	df^a^	Paired *t*-test	*P* value	Cohen *d*	Standard Deviation	N
Odds ratio	26.67	2	4.00	.057	2.309	11.55	3
Clinical significance	13.33	5	1.35	.235	0.551	25.30	6
Forest plots	30.49	60	5.71	.000	0.731	32.17	61
Confidence interval	38.36	54	6.84	.000	0.922	26.00	55

^a^df: degrees of freedom.

### Pretest and Posttest: Comprehension of the Research Concepts

The pretest and posttests had a total n of 124. Eleven individuals participated in the first video-viewing session at CPHA. There were 2 participants for the CI video, 6 participants for the clinical significance video, 1 participant for the forest plot video, and 3 participants for the OR video. From the Eastern Newfoundland workshop, there were an additional 60 participants for the forest plot video, and an additional 53 participants for the CI video.

Pretest and posttest results are summarized in Table 4. Results from paired *t*-tests showed there was an increase in knowledge on forest plots and CIs after watching the URE videos. A statistically significant difference was found for the forest plots’ and the CIs’ pretest and posttest data ([Forest plots: *t*_61_=5.710, *P*<.001, d=0.731], [CIs: *t*_55_=6.835, *P*<.001, d=0.922]). There was a medium-large effect size for the forest plot video pretest and posttest, and a large effect size for the CI video pretest and posttest. Moreover, the OR video pretest and posttest (d=2.309) had a very large effect size, and the clinical significance video pretest and posttest (d=0.551) had a medium effect size. However, statistical significance was not detected for the clinical significance (n=6) and OR (n=3) videos due to small sample sizes.

### Telephone Interviews

Most telephone interview participants (n=18) had watched multiple videos: 15 participants watched the CI video; 12 watched the OR video; 13 watched the clinical significance video; and 12 watched the forest plot video. A summary of the telephone interview results can be found in Textboxes 1-3.

#### Comprehension of Research Concepts

In the telephone interviews, participants stated that the videos acted as a refresher to reinforce concepts they had learned in their Masters’ programs, statistic courses, and continuing education courses. The videos reminded participants of the important points of a research concept. Participants indicated the videos helped them understand how to analyze data to determine whether they were looking at good evidence. One participant noted:

It’s a refresher but it also reminds people how doable it really is. I just thought after seeing it, I thought, this actually makes somebody feel like they could actually just take a pen and paper and do some simple ratios.

**Summary of telephone interview theme 1: increased comprehension of research concepts.**

Main findings:

- URE videos helped participants understand common concepts in research evidence.
- URE videos reinforced important points to know about research concepts.

Quotations:

- “There was a general increase in knowledge and understanding, but I think more importantly for me, there was a reinforcement of what I already knew.” (Participant 1)
- “They may have heard the term, but it’s not something that they use everyday, so we’re just trying to build that capacity within our organization.” (Participant 6)
- “It is a nice refresher. It (URE videos) doesn’t necessarily tell you anything that you didn’t already know, but it reminds you of the important points that sometimes get lost.” (Participant 11)

**Summary of telephone interview theme 2: learning through the videos.**

Subtheme: Delivery of the content

- Main findings
  - Narration and animation in the videos acted as learning aids that supported both visual and auditory learners.
  - URE videos were accessible because they avoided technical jargon.
  - The short length allowed public health professionals to use the videos during their workday.
  - Videos were used both individually and in groups.
- Quotations
  - “I think it (URE Videos) really speaks to all adult learners, you know the ones that like to see things, the ones that like to hear things. I think it speaks to a varied amount of learners.” (Participant 9)
  - “I found that the language that was used was quite clear and plain. The presenter was able to simplify what might be thought of as complex.” (Participant 18)
  - “The length was good. They (URE Videos) weren’t too long, but they were long enough to explain the concept.” (Participant 5)
  - “I can also see these being used at a team meeting. I don’t know if this has been done, but you could pull this (URE Videos) up at a team meeting and say let’s spend the next seven team meetings just do one a week and go over it. What a great way to keep your learning up to date.” (Participant 10)

Subtheme: Content of the videos

- Main findings
  - Examples in the videos were relevant to public health and helped illustrate the key ideas of the research concept.
  - Videos focused on how to interpret a research concept.
- Quotations
  - “I liked how the videos related some of the concepts to practical examples in public health. Giving public health examples we could all relate to made it all the more easier to understand the concept.” (Participant 3)
  - “Some videos I found were more for professionals or decision-makers who want to learn how to interpret some statistical concepts, but some other videos I thought were giving some more information for professionals who would reproduce or would have to make that kind of statistical method…” (Participant 16)
  - “So what’s in it for me is whether a relationship exists, and what the strength of that relationship is between the intervention and the control.” (Participant 14)

**Summary of telephone interview theme 3: supporting the use of research evidence.**

Main findings

- The URE videos helped public health professionals develop core competencies for evidence-informed public health.
- Understanding common research concepts helped public health professionals assess the quality and strength of the research.
- The videos provided a common language to talk about intervention effectiveness.

Quotations

- “One of my main tasks in my job is to make sure that the staff at the health unit have the skills to do evidence-informed public health. So you know, I’m always looking for really good resources that I can pass on to the staff to increase their skill, probably in the appraising stage of articles.” (Participant 8)
- “I got a much better sense of, like if I’m reading a research report or kind of report that is using statistics, I will have a much better sense of what it means and hopefully be able to use it in application.” (Participant 12)
- “It (URE videos) gave me language also to explain it to others in my workplace… I found the videos gave me some clear examples and language that I could help explain it to others.” (Participant 3)

#### Learning Through the URE Videos

##### Delivery of the Content

The telephone interviews revealed that narration and animation in the videos provided learning aids to understand the research concepts covered. One participant thought the videos spoke to all adult learners. The “words, numbers, and pictures” used in the videos appealed to both visual and auditory learners. The narration in the videos helped participants engage with the video content with minimal effort. Participants could just “sit back and listen.” Participants found that the use of clear and plain language helped to explain the technicalities of the research concept, and gave them a clear understanding of the topic. They also found that avoiding technical jargon simplified complicated concepts, making the videos easy to understand. Participants found the videos enjoyable to listen to, and the concepts were accessible to those who were not familiar with research. Participants also thought the narration in the videos made the content easier to understand for auditory learners. They found repeating definitions of the concepts using similar language and having the narrator speak slowly facilitated learning for auditory learners.

Participants, who were visual learners, appreciated the use of animations and visual diagrams. Moreover, participants felt the combination of narration with animations made it easier to follow the content. Participants thought the combination of narration and animation brought the examples used in the videos to life, and made technical research concepts less intimidating. Participants also thought the animations helped to “lighten the gravity of the information” and kept the videos dynamic, which helped maintain viewers’ interest in the content. One participant noted:

The animations were good. They kind of kept you watching and kept you interested and intrigued. They helped you, at certain points, I guess, they helped you understand some of the concepts just viewing the animations, which is not possible sometimes with just having a person talk.

Overall, participants found the videos appropriate and pleasant to watch. They found the videos short and concise, and liked that the videos were quick to watch. They thought that the videos were long enough to explain the concept, but short enough to accommodate interruptions during the workday. The short length of the videos also allowed participants to use the videos as a quick reference.

Participants also noted how the videos could be used individually and in groups to facilitate learning. They felt the videos could be used individually when interpreting the results of a study or in a group. Participants also described how the videos could be used for peer learning in a group setting such as a quick refresher before a meeting, in journal clubs, in classroom settings, or in a larger continuing education program for public health professionals. Most participants preferred watching the videos in a group setting. One participant explained how she used the videos in a meeting:

We reviewed the videos, and then discussed how we would present it to our teammates. What we decided to do was, we brought a laptop and projector into our team meeting, and we showed the videos to our teammates, and then what we did is we talked about it in terms of work that we are currently doing, and we were able to also bring something that we were actually reviewing. So for example, she brought a research article that had a forest plot in it, so we showed that to the team, and explained how this was a real article that was being reviewed. And what I did is, I had been working with our [epidemiology] department, and was currently reviewing health status data. So I brought one of the tables from a health status report that they had prepared for our team. We were able to talk about current and relevant uses and application to the work we were doing.

##### Content of the Videos

The telephone interviews demonstrated that the examples included in the videos were relevant and common topics in public health, which helped participants understand and relate to the content. Participants felt that the examples illustrated what the concept meant and broke down the content into different parts. One participant stated:

By using the examples, it made the definition of odds ratio or whatever I was looking at more real to me, again versus reading it in a textbook. I probably wouldn’t have had as decent of an understanding as I did by having the example, it just brought it more to life for me.

Participants found that all the videos focused on explaining how to interpret a research concept, with some also explaining how to calculate or reproduce a research concept. For example, one participant felt that the video on CIs focused more on the interpretation of the concept, whereas the video on OR explained how to calculate an OR as well as how to interpret it. Participants thought that the videos focused on how to interpret a research concept and were helpful and simpler to follow.

#### Supporting the Use of Research Evidence—From Application to Practice

Participants thought that by increasing the use of research evidence in their practice, they could develop better program interventions and design evaluations to measure the impact of public health initiatives. Participants felt that the URE videos helped public health professionals develop a core competency for evidence–informed decision-making. They felt the videos were effective at building skills in technical research concepts, which allowed them to better engage with research evidence. They also felt that understanding research evidence allows public health professionals to learn about different types of intervention that exist for an issue and why the interventions are effective in particular contexts. One participant expressed:

I really am hoping that our team as we move forward will start to focus more on looking at the type of interventions we do, and making sure that they are research-based and evidence-based, and that would mean we would have to start reading the research more. And I think that’s how this kind of information can help our team. When we’re reading the research, knowing what a confidence interval is, what that means, that way we can better decide if an intervention is something that is worthy of us putting into place or not. I can see us definitely learning from these videos how to better understand the research that has already been done in our field.

Furthermore, participants felt that the URE videos provided them with a deeper understanding of how to interpret and understand the details of research data. One participant noted how understanding common research concepts allowed public health professionals to assess the quality and strength of research evidence. Another participant thought the ability to interpret research evidence could also help public health professionals design evaluations to assess interventions. This participant felt that research and evaluation was important in keeping public health accountable for its action. Another participant stated that a clearer understanding of research concepts could make evaluation less intimidating.

Finally, participants found the videos introduced them to more approachable ways to discuss data by providing a common language to talk about intervention effectiveness. They felt the language to talk about intervention effectiveness would impact the uptake of research evidence in public health work. This participant stated:

It gave me language also to explain it to others in my workplace. We do work with, we do have some students working with us, and it’s always helpful to have a different way to kind of explain a concept, than what you have used in the past. And I found the videos gave me some of those clear examples and language that I could help explain it to others.

## Discussion

### Principal Findings

The Web-based survey and the pretest and posttest demonstrated that the URE videos were effective in increasing public health professionals’ knowledge on ORs, clinical significance, CIs, and forest plots. The results of the survey and pretest and posttest were further supported by the themes uncovered from the telephone interviews. Findings from the telephone interviews demonstrated how the URE videos were an effective continuing professional development resource that builds public health capacity to use research evidence. Public health professionals who participated in this evaluation indicated that they learned how to interpret or understand the research concept covered in the video. They were satisfied with the delivery methods and content of the videos and felt they could apply what they had learned in their professional practice. The evaluation found a relationship between watching the URE video and the ability to recall and comprehend the concepts.

Public health professionals found the content of the URE videos applicable to their work. They thought the URE videos could help them with program implementation and evaluation, because an understanding of research concepts would make research findings and the idea of measurement more approachable. The URE videos also provided the public health professionals with language to talk about data, which increased discussion about research and led to increased engagement with research evidence. Overall, public health professionals were receptive of the URE videos and found the resource to be useful to their work.

The public health workforce is broad. It ranges from medical officers of health to biostatisticians, health promoters, and other positions. The ability to understand common research concepts is a foundational competency for the implementation of effective interventions. Although public health professionals in different roles may use research evidence for different purposes, it is important that all public health professionals have a baseline understanding of common research concepts and the language to meaningfully discuss research evidence with their colleagues. The URE videos can build public health professionals’ capacity to engage with research evidence when planning, implementing, and evaluating public health interventions. This will ensure all public health interventions, from clinical interventions to programming, are effective at improving the health of individuals and communities.

### Limitations

Since the URE videos were only made available on the Internet in 2013, their impact on the practices of public health professionals may not yet be fully known. Future evaluations should further explore the longitudinal impact of the URE videos. Moreover, findings from this evaluation may be limited due to bias from selection and self-assessment. The majority of participants in the Web-based survey were registered NCCMT users. The public health professionals who watched the URE videos may have already be interested and more competent in using research for evidence-informed public health than other public health professionals. However, public health professionals who had not seen the URE videos were asked to watch the videos in order to participate in the telephone interviews. Many of these public health professionals were not familiar with the NCCMT but still addressed the importance of understanding and using research evidence in public health practice. Additionally, self-assessment was used to gather data on participants’ background knowledge on research concepts and whether or not they felt they had gained knowledge from watching the videos. Participants may not have accurately responded to these questions due to distorted self-perceptions.

Furthermore, the external and internal validity of the findings should be considered. The Web-based survey had a low response rate, which may affect the generalizability of the results. Although the survey demonstrated that a medium correlation exists between knowledge level (as determined by knowledge assessment scores) and the clinical significance and OR videos; due to small sample sizes, no statistical significance was detected for the pretest and posttest results for the two videos. Similarly, a medium-large effect size was detected in the pretest and posttest for the forest plot video; however, a statistical significant correlation between knowledge level and the forest plot video was not detected in the Web-based survey. Additional testing of the clinical significance, OR, and forest plot videos need to be conducted to gather more consistent results. Also, none of the knowledge assessment questions in the Web-based survey or pretest and posttest were piloted due to time restraints. It is unknown whether the questions adequately captured the constructs needed to understand the OR, clinical significance, CI, and forest plot concepts. Finally, Web-based survey and telephone participants had the option of watching the videos in French or English. However, data was not collected on the language used to watch the videos.

Future evaluation of the URE videos should include the following: test of the validity of the knowledge assessment questions, increase in sample sizes and the number of knowledge assessment questions asked for each video (to increase the statistical significance of the findings), and a determination of whether knowledge gained differed when watching the videos in French versus English. Despite the limitations of the evaluation, the findings provided useful insights into public health professionals’ experience with the URE videos, and the manner in which they intend to use the videos in their practice.

### Future Applications

Since public health is a broad multidisciplinary field, wide-reaching and targeted continuing education strategies are necessary to facilitate universal public health competencies [14,15]. Continuing professional development in public health aims to maintain core professional competencies for public health practitioners. The ability to interpret research evidence is a core competency for evidence-informed decision making [6,8,10]. Bridging knowledge gaps in understanding research concepts and interpreting research evidence can address barriers to evidence-informed decision making in public health.

Continuing professional development training provides the knowledge and skills necessary to improve population health [14]. Public health professionals are motivated to participate in continuing professional development activities due to peer pressure, to maintain a high standard of professional competence, and because they desire self-advancement [16]. However, educational strategies and technology used to deliver continuing professional development need to be considered to facilitate effective learning within the constraints of professional practice.

Web-based technology is becoming a popular way to administer continuing professional education in public health. According to a study conducted by the American Public Health Association [17], 76% of public health professionals prefer continuing education training to be delivered through webcasts and webinars, and 65% prefer Web-based, self-paced training. The Centers for Disease Control and Prevention in the United States (Public Health Training Network), the United Kingdom’s Department of Health (Teaching Public Health Networks), and Canada’s Public Health Agency of Canada (Skills Online) have all created Web-based continuing professional development training programs for public health professionals [10,18-20]. However, there are still a limited number of free, open-access learning resources on the Web for public health [21].

Effective Web-based continuing education programs should use appropriate delivery methods and consider the type of information being provided [19]. Public health professionals were satisfied with the delivery and content of the videos. They liked the URE videos because the combination of animation, narration, and plain language made the content clear. Enjoyment in learning can motivate learners and allow them to relax to better engage with the content [22]. Moreover, time is a major barrier to engaging in evidence-informed public health [7]. The videos were short enough to fit into existing work schedules.

According to Friedman [23], effective continuing education programs should appeal to a range of learners and consider the needs of individuals with different learning styles to support learners to be proactive in their learning. The videos were delivered using strategies that targeted different types of professional adult learners. The URE videos addressed auditory, visual, independent, and group learners. The fact that these videos were dynamic and included animation, examples, and narration may have facilitated learning of the concepts.

The URE video series add to the existing public health professional development resources by offering specific training on understanding common research concepts that are freely accessible on the Internet. Since the completion of this evaluation, additional videos have been created on the following topics: how to find research evidence, the evidence-informed decision making framework, types of reviews, relative risk, number needed to treat, *P* values, and the standardized mean difference [11]. Public health professionals who participated in this evaluation suggested additional topics they would like to see covered in the URE Web-based video series, which included tests for heterogeneity, statistical significance, and effect sizes. Participants also suggested that future videos could cover how to understand qualitative and mixed-methods research.

### Conclusions

The URE video series is one of the many resources that the NCCMT offers to build capacity for evidence-informed public health. The lack of skill in understanding common research concepts was identified as a barrier in engaging with evidence-informed decision making. Public health professionals need to be able to understand research evidence to be able to use it in their practice.

Public health professionals’ knowledge of common research concepts was impacted by the URE videos. The delivery and content of the URE videos helped to facilitate learning. An increased understanding of research concepts made public health professionals feel that they could use research evidence to inform program implementation, and made program evaluation more approachable; it also gave them the language to discuss data and research with their colleagues. Overall, the URE videos were received well, and public health professionals hoped for more videos on different research topics in the future. The NCCMT will continue to expand and develop the Web-based URE video series and other resources to build capacity in evidence-informed decision making among Canadian public health professionals, and empower them to use the best available evidence when making decisions to improve community and population health outcomes.

## Appendix

Multimedia Appendix 1 Web-based survey and pretest/posttest knowledge assessment questions.

## Abbreviations

CPHA: Canadian Public Health Association.
NCCMT: National Collaborating Centre for Methods and Tools.
URE: Understanding Research Evidence.
