# Supplementary material for: How the “Understanding Research Evidence” Web-Based Video Series From the National Collaborating Centre for Methods and Tools Contributes to Public Health Capacity to Practice Evidence-Informed Decision Making: Mixed-Methods Evaluation
Source: J Med Internet Res. 2017 Sep 28;19(9):e286. doi: 10.2196/jmir.6958 (PMC5639207; doi:10.2196/jmir.6958)
Supplement: Multimedia Appendix 1 [file jmir_v19i9e286_app1.pdf]

Multimedia Appendix 1:

Web-based Survey and Pretest and Posttest Knowledge Assessment Questions

1 Web-based Survey Knowledge Assessment Questions

The following questions were included in the web-based survey. The questions also assessed participants' knowledge of the concepts covered in the URE videos on odds ratio, clinical significance, forest plots, and confidence intervals.

Clinical Significance

1. What is clinical significance?
    - **a. Whether the effect found in the study is big enough to justify investing in the intervention**
    - b. When an intervention only works in a clinical setting
    - c. Confidence that the results are real
    - d. The confidence interval in a study

2. What is the most relevant information you need to calculate clinical significance? Select all that apply.
    - a. Professional judgment
    - b. Effect size
    - c. Confidence intervals
    - **d. Both a and c**

Odds Ratios

1. What does an odds ratio tell you?
    - a. Whether or not an intervention works
    - b. The sample population size compared to the general population
    - c. Odds that an action/intervention will lead to a particular outcome
    - **d. Both a and c**

2. What does an odds ratio greater than 1 mean?
    - **a. The outcome of interest is more likely in the intervention group.**
    - b. The outcome of interest is less likely in the intervention group

Confidence Intervals

1. What is a confidence interval?
    - a. Indicates range of intervention effectiveness
    - b. Indicates consistency of the results of the study to the results for a hypothetical sample
    - c. Indicates we can be 95% certain of results of an individual study
    - d. Both a and b

2. What is the interpretation of the result when a confidence interval includes the number of no effect?

   a. **The study results of an intervention may be due to chance and may not be real**
   b. The range of intervention effectiveness is broadened
   c. The effect size in the study will be large enough to justify investing in the intervention
   d. It will be more difficult to decide whether or not to implement an intervention

3. How does a confidence interval help you determine whether or not to apply an intervention?

   a. Helps determine how confident you can be in the results
   b. Describes the effects an intervention has across various samples of the population, not just the effect for one sample
   c. Helps determine statistical and clinical significance
   d. **All of the above**

Forest Plots

1. What does a forest plot tell you?

   a. The range of intervention effects from the individual studies
   b. Whether the intervention is statistically significant
   c. The overall effect size of the intervention
   d. **All of the above**

2. How can you determine the range of effects an intervention can have from a forest plot?

   a. The vertical points of the diamond
   b. The outcome measures on the horizontal axis
   c. **The left and right points of the diamond**
   d. The individual points in the forest plot

2 Pretest and Posttest Knowledge Assessment Questions

The following questions were included in the pretest and posttest. The questions assessed participants' knowledge of the concepts covered in the URE videos on odds ratio, clinical significance, forest plots, and confidence intervals.

Odds Ratios

1. The odds ratio tells you whether or not an intervention works, and the effect size of the intervention.
   a. **True**
   b. False

2. What does an odds ratio greater than 1 mean?
   c. **The outcome of interest is more likely in the intervention group.**
   d. The outcome of interest is less likely in the intervention group.

3. What does an odds ratio of 1 mean?
    a. The intervention had no effect
    b. The outcome of interest is less likely in the intervention group

4. In this hypothetical study, the effectiveness of a citywide health promotion text-message intervention to increase helmet-use in daily bike commuters is being tested.

   What is the odds ratio in this scenario?

	Intervention (Received text messages)	Control (Did not receive text messages)
Used helmets	16	16
Did not use helmets	2	4

   a. 0.5
   b. 2
   c. 0.25
   d. 1

5. In a hypothetical study on the effect of a health promotion intervention to encourage low-risk drinking practices for women, the odds ratio is 0.87.
   Which of the following statements correctly interprets this finding?

   a. Women who received the health promotion intervention were more likely to practice low-risk drinking
   b. Women who received the health promotion intervention were less likely to practice low-risk drinking
   c. Women who did not receive the health promotion intervention were less likely to practice low-risk drinking
   d. The health promotion intervention had no effect on women’s low-risk drinking practices

Clinical Significance

1. What is clinical significance?
   a. Whether the effect found in the study is big enough to justify investing in the intervention
   b. When an intervention only works in a clinical setting
   c. The effect found in the study is real
   d. The confidence interval in a study

2. What is the most relevant information you need to calculate clinical significance? Select all that apply.
    a. **Professional judgment**
    b. Effect size
    c. Odds ratios/relative risk
    d. **Confidence intervals**

3. What factors determine clinical significance? Select all that apply.
    a. **The resources available to the practitioner**
    b. **The range of intervention effect sizes stated by the confidence interval in the research evidence**
    c. The intervention effect size determined by the odds ratios/relative risks in the research evidence
    d. The characteristics of the sample population in the research evidence

4. In a hypothetical example, you find a systematic review that found that health promotion text-messages were very effective in increasing bike helmet use amongst bike commuters in the city. The study showed that there was a **50-80%** increase in bike helmet use after exposure to the text-message intervention.

   When further exploring this intervention, you find text-messages are not included in many cellphone plans in your city. The health promotion text messages would cost bike commuters in your city $0.75 for each text message they receive.

   In this situation, are health promotion text-messages a clinically significant intervention?
    a. Yes
    b. **No**
    c. Not enough information provided to determine the answer

5. In another hypothetical example, you find a meta-analysis that found that a snow shoveling and salting program reduces hip injuries in seniors by **90%.**

   You have decided that you can design a program that pairs volunteers with seniors to help them shovel and salt their walkways and sidewalks when it snows.

   In this situation, is the snow-shoveling and salting a clinically significant intervention?
    e. Yes
    f. No
    g. **Not enough information provided to determine the answer**

Confidence Intervals

1. The confidence interval shows the range of effects an intervention can have, if the same study was replicated 100 times.
    a. **True**
    b. False

2. Is it necessary to consider both p-values and confidence intervals?
    a. Yes
    b. No

3. How can you tell if the finding in a study is statistically significant by looking at the 95% confidence interval?

    a. The finding is statistically significant if the 95% confidence interval includes the relative risk or odds ratio in its range.
    b. The finding is statistically significant if the 95% confidence interval includes the number of no effect (1) in its range, and the relative risk/odds ratio is above 1
    c. The finding is statistically significant if the 95% confidence interval does not include the number of no effect (1) in its range.
    d. The finding is statistically significant if the 95% confidence interval does not include the number of no effect (1) in its range, and the relative risk/odds ratio is above 1.

4. What can the confidence interval tell you about an intervention?
    a. The effect the intervention will have on a specific population
    b. The different outcomes from an intervention
    c. The statistical significance of an intervention
    d. The clinical significance of an intervention

5. In a hypothetical example of a text-message intervention to increase helmet use amongst daily bike commuters, if the confidence interval indicates an effect between a 10% reduction in bike helmet use and a 60% increase in bike helmet use. Would you implement the text-message intervention?
    a. Yes
    b. No

Forest Plots

1. What does a forest plot tell you? Select all that apply
    e. The range of intervention effects from the individual studies
    f. Whether the intervention is statistically significant
    g. The overall effect size of the intervention
    h. All of the above

2. How can you determine the range of effects an intervention can have from a forest plot?
    e. The vertical points of the diamond
    f. The outcome measures on the horizontal axis
    g. The left and right points of the diamond
    h. The individual points in the forest plot

3. Using a forest plot, what is a quick way to determine if the intervention had no effect on the outcome of interest?
   a. If the individual points are clustered on the left side of the forest plot
   b. If the individual points are clustered on the right side of the forest plot
   c. If the individual points are clustered around the vertical line
   d. If the left and right points of the diamond touches or intersects the vertical line

4. Imagine you are reading a meta-analysis on the effect of a health promotion text-message intervention on bike helmet use amongst daily bike commuters. Based on this hypothetical forest plot, is this intervention effective?
   a. Yes
   b. No

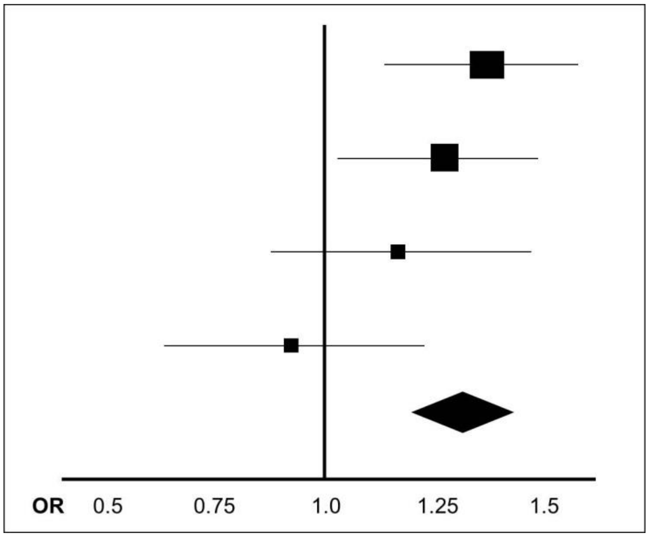

5. From this hypothetical forest plot determine how many studies within the meta-analysis found that text-message intervention reduced bike helmet use amongst daily bike commuters.
   a. 1
   b. 3
   c. 4
   d. Not enough information provided

(Association of Faculties of Medicine in Canada, 2014)
